# Reliability Evaluation Based on Different Distributions of Random Load

**DOI:** 10.1155/2013/415327

**Published:** 2013-10-02

**Authors:** Peng Gao, Liyang Xie

**Affiliations:** ^1^Department of Chemical Mechanical Engineering, Liaoning Shihua University, Liaoning 113001, China; ^2^Department of Mechanical Engineering and Automation, Northeastern University, Liaoning 110004, China

## Abstract

The reliability models of the components under the nonstationary random load are developed in this paper. Through the definition of the distribution of the random load, it can be seen that the conventional load-strength interference model is suitable for the calculation of the static reliability of the components, which does not reflect the dynamic change in the reliability and cannot be used to evaluate the dynamic reliability. Therefore, by developing an approach to converting the nonstationary random load into the random load whose pdf is the same at each moment when the random load applies, the reliability model based on the longitudinal distribution is derived. Moreover, through the definition of the transverse standard load and the transverse standard load coefficient, the reliability model based on the transverse distribution is derived. When the occurrence of the random load follows the Poisson process, the dynamic reliability models considering the strength degradation are derived. These models take the correlation between the random load and the strength into consideration. The result shows that the dispersion of the initial strength and that of the transverse standard load coefficient have great influences on the reliability and the hazard rate of the components.

## 1. Introduction

Reliability is an important index to the design, manufacture, and maintenance of products. Products failure when environment load is higher than strength. Reliability is defined as the probability that the products function successfully in the mission duration. The well-known load-strength interference (LSI) model plays a significant role in the analysis of the reliability of the mechanical components and systems. 

**Figure 1:**
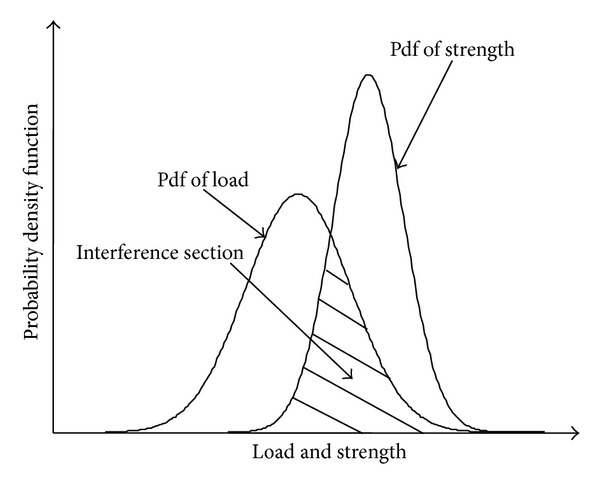
The schematic illustration of the LSI model.

In the LSI model, the environment load and the strength are random variables. The probability density function (pdf) of the load and that of the strength are shown in Figure 1. The interference section of the curves indicates that components may fail when the load exceeds the strength. The LSI model is used to deal with the static reliability problems, which assumes that the load is independent of the strength [1–5]. In practice, however, mechanical components always operate under random load, and the strength of the components degrades due to the repeated application of the random load. Therefore, both the load and the strength should be considered as the stochastic processes, and it is necessary to develop dynamic reliability models.

Some efforts have been made to investigate the dynamic reliability models based on the LSI model. Wen and Chen [6] analyzed reliability of the mechanical structure under the time varying loads. Lewis and Chen [7] developed a dynamic reliability model and analyzed the failure rate of the components. Enright and Frangopol [8] proposed an approach to calculating the reliability, in which the load and the strength are time variant quantities. Huang and Askin [9] generalized the LSI model and took the strength degradation into account. Ahammed and Melchers [10] analyzed the reliability of structures under multiparameter time-varying load in the space of the load. Xue and Yang [11] developed a dynamic reliability model based on the improved LSI model. van Noortwijk et al. [12] developed a reliability model and the load and the strength are described as two stochastic processes. Blanchart [13] established a failure model based on a physical LSI model. Xue and Yang [14] analyzed the upper and lower bounds of a dynamic reliability model.

The models in these papers held the hypothesis that the strength was independent of the load and was described as a simple function of time. Besides, the pdf of the random load was assumed to be the same at each moment when the random load applies. However, when strength degradation is taken into account, the strength is the function of the occurrence frequency of the random load and the magnitude of the random load, especially for mechanical components. It may cause errors in the calculation of reliability to neglect the correlation between the random load and the strength. Moreover, in order to develop the dynamic reliability model, further analysis should be made to obtain the relationship between the strength and the time, instead of assuming a simple function of time to describe the strength. 

Furthermore, different distributions of random load can be obtained according to different statistic methods. Therefore, it is necessary to define and distinguish the distributions of the random load, which may otherwise lead to the misusage of the reliability models. In addition, components are subject to nonstationary random load sometimes. In such a situation, great differences may exist in the mean value and the autocorrelation function among different samples of the random load, which makes it difficult to satisfy the ergodic conditions. Thus, the pdf of the random load is different at each moment when the random load applies, and it is important to develop an easy-to-use approach to calculating the reliability of the components under the nonstationary random load.

In this paper, we define the distributions of the random load according to different statistic methods. Then, the dynamic reliability models considering the correlation between the random load and the strength are derived based on the LSI model.

## 2. Analysis of the Distribution of Random Load

**Figure 2:**
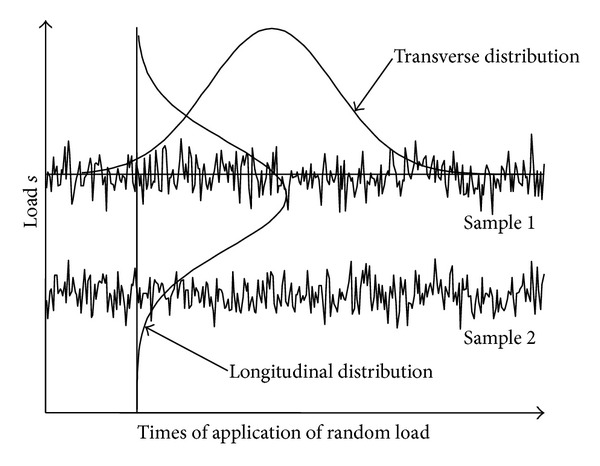
The schematic illustration of the distributions of the random load.

In order to obtain the statistic characteristics of the random load, the samples of the random load are recorded as shown in Figure 2. According to different statistic methods to dealing with the samples of the random load, we define the distribution of the random load at each moment as the longitudinal distribution, while the distribution acquired from each sample of the random load is defined as the transverse distribution. 

From the definition of the distribution of the random load, it is easy to see that the longitudinal distribution reflects the probabilities that the loads with different amplitudes occur at a determinate moment, while the transverse distribution reflects the proportion between the occurrence frequencies of the loads with different magnitudes in the mission duration. Most reliability models are based on the longitudinal distribution of the random load. Besides, when the pdf in the LSI model is the longitudinal pdf, the LSI model actually calculates the reliability when the random load applies once. When the pdf in the LSI model is the transverse pdf, the LSI model calculates the reliability when the strength does not degrade.

 Provided that the longitudinal pdf at each moment when the random load applies is the same, it is reasonable to assume that the transverse pdf from each sample is the same, which is approximatively identical with the longitudinal pdf. However, the random load is nonstationary sometimes, and great differences may exist in the mean value and the autocorrelation function among different samples of the random load, which makes it difficult to satisfy the ergodic conditions. In this situation, the longitudinal pdf is different at each moment when the random load applies and cannot be obtained from the transverse pdf. Thus, when the number of the samples of the random load is large, a convenient method to calculate the reliability of the components under the nonstationary random load based on the longitudinal pdf will be proposed in the following section. When the number of the samples of the random load is small, only a few transverse pdfs can be obtained from the samples, and it is impossible to obtain the longitudinal pdf. Then the reliability models based on the transverse distribution under the nonstationary random load will be developed in this paper. 

## 3. Reliability Model Based on the Longitudinal Distribution 

Suppose that the initial reliability is *R*_0_. The longitudinal pdf of the load and the pdf of the strength at the moment when the random load applies for the *i*th time are denoted as *f*_*s*_*i*__(*s*_*i*_) and *f*_*r*_*i*__(*r*_*i*_), respectively. Provided that the random load has applied for *n* times, the reliability of the components can be calculated as

(1) $$\begin{array}{c} R\left(n\right)=R_{0}\overset{n}{\underset{i=1}{\prod }}\overset{\infty }{\underset{-\infty }{\int }}f_{s_{i}}\left(s_{i}\right)\overset{\infty }{\underset{s_{i}}{\int }}f_{r_{i}}\left(r_{i}\right)dr_{i}\;ds_{i}. \end{array}$$

For nonstationary random load, the longitudinal pdf at each moment when the load applies is different. Moreover, it is impossible to obtain the longitudinal distribution of the random load at each moment by tests, which means a daunting amount of work. Besides, the randomness of the load makes it difficult to describe the degradation process of the strength. Therefore, a new approach to converting the nonstationary random load into the random load whose pdf is the same at each moment when the random load applies is proposed based on the Miner damage accumulation rule as follows.

According to the Miner damage accumulation rule, the damage caused by a determinate load *S* for once is

(2) $$\begin{array}{c} D_{S}\left(1\right)=\frac{1}{N_{S}}, \end{array}$$

where *N*_*S*_ is the lifetime of the components under *S*. Moreover, according to the *S*-*N* curves of the components, it can be obtained that

(3) $$\begin{array}{c} N_{S}S^{m}=C. \end{array}$$

Hence, the mean value of the damage caused by the application of the random load for once can be expressed as

(4) $$\begin{array}{c} E\left(D_{S}\left(1\right)\right)=\frac{1}{C}\overset{\infty }{\underset{-\infty }{\int }}s^{m}f_{s}\left(s\right)ds. \end{array}$$

The damage in (4) equals that caused by an equivalent load *s*_*q*_ for once. According to (4), *s*_*q*_ can be expressed as

(5) $$\begin{array}{c} s_{q}={\left(\overset{\infty }{\underset{-\infty }{\int }}s^{m}f_{s}\left(s\right)ds\right)}^{1/m}. \end{array}$$

Suppose the equivalent load at the moment when the random load applies for the *j*th time is *s*_*qj*_ (*j* = 1,2,…, *n*) and the pdf of *s*_*q*_ is *f*_*s*_*q*__(*s*_*q*_). We define *s*_0_ as the longitudinal standard load, which can be written as

(6) $$\begin{array}{c} s_{0}={\left(\overset{\infty }{\underset{-\infty }{\int }}s_{q}^{m}f_{s_{q}}\left(s_{q}\right)ds_{q}\right)}^{1/m}. \end{array}$$

According to the Miner damage accumulation rule and (4), the damage caused by *s*_*qj*_ for once is equal to that caused by *s*_0_ for *β*_*j*0_ times, which can be expressed as

(7) $$\begin{array}{c} \frac{s_{qj}^{m}}{C}=\beta_{j0}\frac{s_{0}^{m}}{C}. \end{array}$$

From (7), *β*_*j*0_ can be obtained as follows:

(8) $$\begin{array}{c} \beta_{j0}={\left(\frac{s_{qj}}{s_{0}}\right)}^{m}. \end{array}$$

According to (7), it is easy to prove that the mean value of *β*_*j*0_ is 1. Therefore, the nonstationary random load can be approximated by an equivalent random load, whose longitudinal pdf at each moment when the load applies is identical, and the equivalent load at each moment equals the longitudinal standard load *s*_0_. Consequently, the reliability of the components under nonstationary random load can be approximately calculated as follows.

(1) Suppose that the total times of application of the random load in the period of test is *N*. Divide *N* into *n*_*t*_ small subintervals and obtain the longitudinal pdf at a moment when the load applies in each subinterval. Then, calculate the mean values of the random load at the selected *n*_*t*_ moments that are denoted as *m*_*j*_ (*j* = 1,2,…, *n*_*t*_).
(2) According to (5), calculate the equivalent loads at the selected *n*_*t*_ moments and the mean value of *m*_*j*_ (*j* = 1,2,…, *n*_*t*_) that can be expressed as
  (9) $$\begin{array}{c} m_{s}=\frac{1}{n_{t}}\overset{n_{t}}{\underset{j=1}{\sum }}m_{j}. \end{array}$$
(3) Calculate the longitudinal standard load *s*_0_ as follows:
  (10) $$\begin{array}{c} s_{0}={\left(\frac{1}{n_{t}}\overset{n_{t}}{\underset{j=1}{\sum }}s_{qj}^{m}\right)}^{1/m}. \end{array}$$
(4) Determine a normal distributed pdf with the mean value of *m*_*s*_ and the *m*-order original moment of *s*_0_^*m*^, which is denoted as *f*_*s*_0__(*s*).
(5) Calculate the reliability of the components as follows:
  (11) $$\begin{array}{c} R\left(n\right)=R_{0}\overset{n}{\underset{i=1}{\prod }}\overset{\infty }{\underset{-\infty }{\int }}f_{s_{0}}\left(s\right)\overset{\infty }{\underset{s}{\int }}f_{r_{i}}\left(r_{i}\right)dr_{i}\;ds. \end{array}$$

This is the method to evaluate the reliability of the components under the nonstationary random load based on the longitudinal distribution. In practice, strength is the function of the times of load application and the magnitude of the load. It might cause errors in the analysis of the reliability to neglect the correlation between the random load and the strength. In general, the strength can be expressed as [15]

(12) $$\begin{array}{c} r\left(n\right)=r_{0}{\left(1-D\left(n\right)\right)}^{\alpha }, \end{array}$$

where *r*_0_ and *α* are the initial strength and the material parameter, respectively. *D*(*n*) is the cumulative damage that is determined by the times of load application and the magnitude of the load. As mentioned above, the equivalent load is the longitudinal standard load *s*_0_. Therefore, according to the Miner damage accumulation rule, (13) can be written as

(13) $$\begin{array}{c} r\left(n\right)=r_{0}{\left(1-\frac{ns_{0}^{m}}{C}\right)}^{\alpha }. \end{array}$$

When the initial strength is a random variable, the pdf of strength after arbitrary times of application of the random load can be obtained from (13), which is denoted as *f*_*r*_*i*_|*s*_0__(*r*_*i*_) (*i* = 1,2,…, *n*). Therefore, (11) can be simplified as

(14) $$\begin{array}{c} R\left(n\right)=R_{0}\overset{n}{\underset{i=1}{\prod }}\overset{\infty }{\underset{-\infty }{\int }}f_{s_{0}}\left(s\right)\overset{\infty }{\underset{s}{\int }}f_{r_{i}|s_{0}}\left(r_{i}\right)dr_{i}\;ds. \end{array}$$

As a matter of fact, it is difficult to obtain the statistical characteristics of the nonstationary random load through tests. Besides, the evaluation of the reliability of the components under nonstationary considering the correlation between the random load and the strength is even more difficult. This paper provided an approximate approach to dealing with these problems. 

## 4. Reliability Model Based on Transverse Distribution

As described above, the distribution obtained from each sample of the random load is a transverse distribution. The transverse distribution reflects the proportion between the occurrence frequencies of the loads with different magnitudes in the mission duration. Owing to the limitations in the economy, time, or equipments, engineers can obtain only a few samples of the nonstationary random load sometimes. In this situation, it is impossible to acquire the longitudinal distribution of the random load. Therefore, it is necessary to establish the reliability models based on the transverse distribution of the random load. 

Suppose that there is a transverse distribution from a sample of the random load that is denoted as *f*_1_(*s*). According to the meaning of the transverse distribution, when the total times of load application are *n*, the occurrence frequency of the load with the magnitude of *S*_1_ is

(15) $$\begin{array}{c} n_{1}=nf_{1}\left(S_{1}\right)ds. \end{array}$$

The total damage caused by the random load that applies for *n* times is

(16) $$\begin{array}{c} D_{t}\left(n\right)=\frac{n}{C}\overset{\infty }{\underset{-\infty }{\int }}s^{m}f_{1}\left(s\right)ds. \end{array}$$

Provided that there exists a equivalence load denoted as *s*_*tq*_, which applies for *n* times and causes the same damage, according to (16), *s*_*tq*_ can be expressed as

(17) $$\begin{array}{c} s_{tq}={\left(\overset{\infty }{\underset{-\infty }{\int }}s^{m}f_{1}\left(s\right)ds\right)}^{1/m}. \end{array}$$

For the limited samples of the random load, we can first obtain the transverse pdfs of them and calculate the equivalent loads of them. Then we can obtain the pdf of the equivalent loads denoted as *f*_*tq*_(*s*). Define the transverse standard load as follows:

(18) $$\begin{array}{c} s_{t}={\left(\overset{\infty }{\underset{-\infty }{\int }}s^{m}f_{tq}\left(s\right)ds\right)}^{1/m}. \end{array}$$

For a given sample of the random load, suppose that its transverse pdf is *f*_*t*_1__(*s*), whose equivalent load is *s*_*tq*1_. According to the damage equivalence method as described above, the effect when the random load applies for *n* times is equivalent to that when *s*_*tq*1_ applies for *n* times. Besides, it is also equivalent to that when the transverse standard load *s*_*t*_ applies for *n*_2_ times that can be calculated as follows:

(19) n2=n∫−∞∞smstmft1(s)ds=nstq1mstm.

For all the given samples of the random load, we define the transverse standard load coefficient *k* as

(20) $$\begin{array}{c} k=\frac{s_{tq}^{m}}{s_{t}^{m}}. \end{array}$$

The pdf of *k* can be derived from *f*_*tq*_(*s*) as

(21) $$\begin{array}{c} f_{k}\left(k\right)=f_{tq}\left(s_{t}k^{1/m}\right)\left|\frac{s_{t}}{m}k^{\left(1-m\right)/m}\right|. \end{array}$$

It should be noted that one of the most important properties of *k* is that its mean value is 1, which can be proved as follows:

(22) E(k)=E(stqmstm)=∫−∞∞stqmstmftq(s)ds=1.

Therefore, the reliability of the components based on the transverse distribution can be calculated as follows:

(23) $$\begin{array}{c} R\left(n\right)=R_{0}\overset{\infty }{\underset{-\infty }{\int }}f_{k}\left(k\right)\left(\overset{n\times k}{\underset{i=1}{\prod }}\left(\overset{\infty }{\underset{s_{t}}{\int }}f_{r_{i}|s_{t}}\left(r\right)dr\right)\right)dk, \end{array}$$

where *f*_*r*_*i*_|*s*_*t*__(*r*) denotes the pdf of the strength when the transverse standard load *s*_*t*_ applies for the *i*th time and can be obtained according to (13). 

## 5. Dynamic Reliability Models

In this section, the dynamic reliability models are developed when the occurrence of the load follows the Poisson process. The Poisson process can be used to describe the times of the application of the load with the intensity *λ*(*t*), and the probability that the load applies for *n* times in time *t* is

(24) Pr[n(t)−n(0)=n]  =(∫0tλ(t)dt)nn!exp⁡(−∫0tλ(t)dt).

### 5.1. Dynamic Reliability Model Based on the Longitudinal Distribution

Suppose that *n*(0) = 0. According to the total probability formula, (14) and (24), the reliability of the components in time *t* can be expressed as

(25) R(t)=∑k=0∞P(n(t)=k)Rk=exp⁡(−∫0tλ(t)dt) +∑n=1∞(∫0tλ(t)dt)nn!exp⁡(−∫0tλ(t)dt)    ×(∏i=1n∫−∞∞fs0(s)∫s∞fri|s0(ri)dri ds),

where *R*_*k*_ is the reliability after the random load applies for *k* times. From (25), the hazard rate of the component can be derived as

(26) $$\begin{array}{c} \beta \left(t\right)=-\frac{R^{\prime }\left(t\right)}{R\left(t\right)}=\frac{a_{1}}{a_{2}}, \end{array}$$

where

(27) a1=λ(t)[1−∑n=1∞(∫0tλ(t)dt)nn!       ×(n∫0tλ(t)dt−1)       ×(∏i=1n∫−∞∞fs0(s)∫s∞fri|s0(ri)dri ds)],a2=1+∑n=1∞(∫0tλ(t)dt)nn!     ×(∏i=1n∫−∞∞fs0(s)∫s∞fri|s0(ri)dri ds).

**Figure 3:**
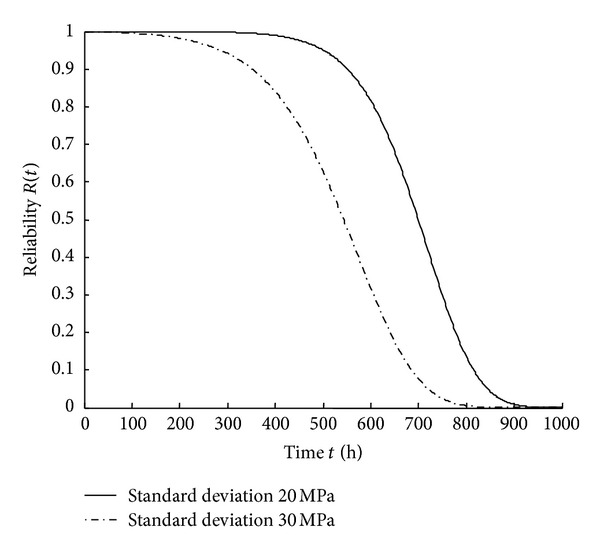
Reliability curves with different standard deviations of *r*_0_.

**Figure 4:**
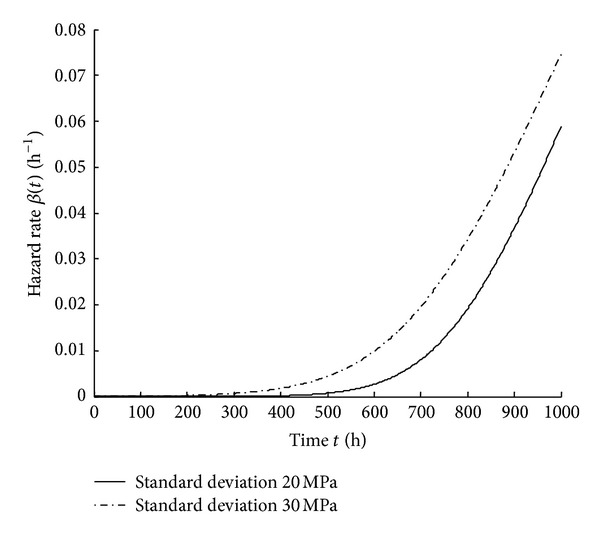
Hazard rate curves with different standard deviations of *r*_0_.

From (25), it is easy to see that the reliability increases as the mean value of *r*_0_ increases. In the following, the influences of the dispersion of *r*_0_ on the reliability and the hazard rate of the components will be analyzed. Suppose that the random load follows the Poisson process with the intensity of 0.6 h^−1^, and *C* = 7.5 × 10^6^, *α* = 1, and *m* = 2. The mean value of the load with the equivalent distribution is 458 MPa., and the longitudinal standard load is 500 MPa. The initial strength *r*_0_ follows the *s*-normal distribution with the mean value of 600 MPa. When the standard deviation of *r*_0_ is 20 MPa and 30 MPa, respectively, the reliability and the hazard rate are shown in Figures 3 and 4.

From Figures 3 and 4, it can be seen that reliability decreases and hazard rate increases as time increases. Moreover, the dispersion of initial strength has an influence on the reliability and the hazard rate. When the dispersion of the initial strength is larger, the reliability is lower and the hazard rate becomes higher.

### 5.2. Dynamic Reliability Model Based on the Transverse Distribution

According to the total probability formula, (23) and (24), the reliability of the components in time *t* can be expressed as

(28) R(t)=exp⁡(−∫0tλ(t)dt) +∫−∞∞fk(k)[∑n=1∞(∫0tλ(t)dt)nn!exp⁡(−∫0tλ(t)dt)         ×(∏i=1n×k(∫st∞fri|st(r)dr))]dk.

From (28), the hazard rate of the component can be derived as

(29) $$\begin{array}{c} \beta \left(t\right)=-\frac{R^{\prime }\left(t\right)}{R\left(t\right)}=\frac{a_{3}}{a_{4}}, \end{array}$$

where

(30) a3=λ(t) −∫−∞∞fk(k)[∑n=1∞(∫0tλ(t)dt)n−1(n−1)!λ(t)         ×(1−∫0tλ(t)dtn)         ×(∏i=1n×k(∫st∞fri|st(r)dr))]dk,a4=1 +∫−∞∞fk(k)[∑n=1∞(∫0tλ(t)dt)nn!         ×(∏i=1n×k(∫st∞fri|st(r)dr))]dk.

**Figure 5:**
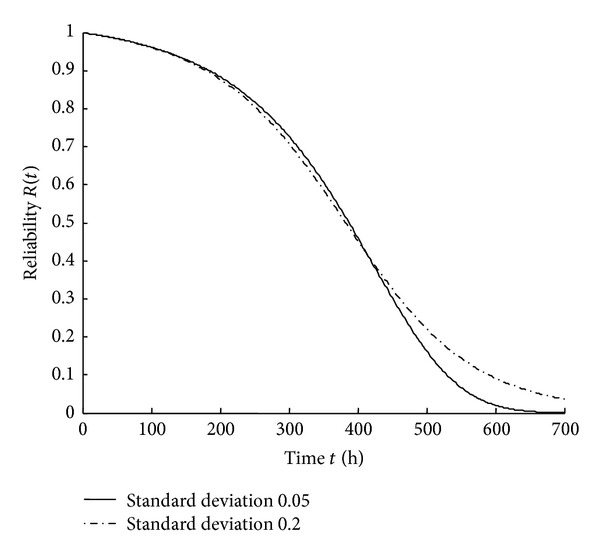
Reliability curves with different standard deviations of *k*.

**Figure 6:**
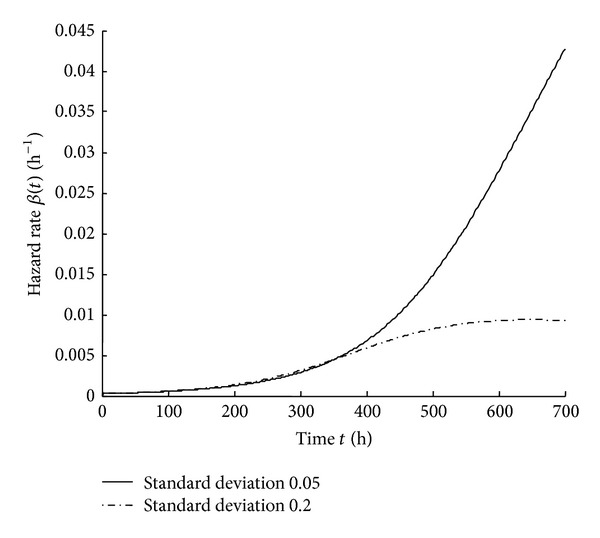
Hazard rate curves with different standard deviations of *k*.

As a matter of fact, the initial strength has the same influences on the reliability and the hazard rate calculated by (28) and (29) as that on the reliability, and the hazard rate calculated according to (25) and (26). In addition, the mean value of the transverse standard load coefficient is 1. Therefore, in the following, we will focus on the influences of the dispersion of the transverse standard load coefficient on the reliability and the hazard rate. Suppose that the components operate under the random load, of which the occurrence follows the Poisson process. The intensity of the Poisson process is 0.6 h^−1^. The transverse standard load is 100 MPa and *C* = 10^7^, *α* = 1, and *m* = 2. The initial strength *r*_0_ follows the *s*-normal distribution with the mean value of 200 MPa and the standard deviation of 20 MPa. Assume that the transverse standard load coefficient *k* follows the *s*-normal distribution with the mean value of 1. When the standard deviation of *k* is 0.05 and 0.2, respectively, the reliability and the hazard rate are shown in Figures 5 and 6.

From Figures 5 and 6, it can be seen that reliability decreases and hazard rate increases as time increases. In the initial failure period and random failure period, when the dispersion of the transverse standard load coefficient is smaller, the reliability is higher and the hazard rate is lower. In the wear-out period, the reliability and the hazard rate are quite sensitive to the dispersion of the transverse standard load coefficient. When the dispersion of the transverse standard load coefficient is larger, the reliability is higher and the hazard rate is lower. This property of the transverse standard load coefficient can be used to provide information for the maintenance and replacement of the components.

## 6. Conclusions 

The reliability models of the components under the nonstationary random load are developed in this paper. At first, the distributions of the random load are defined. Then the dynamic reliability model based on the longitudinal distribution of the random load is derived. When constructing the reliability model based on the longitudinal distribution of the random load, an approach to converting the nonstationary random load into the random load whose pdf is the same at each moment when the random load applies is proposed. The results show that larger dispersion of the initial strength makes the reliability decrease faster and the hazard rate increase faster. 

Furthermore, by defining the transverse standard load and the transverse standard load coefficient, the dynamic reliability model based on the transverse distribution is derived. The results show that the dispersion of the transverse standard load coefficient has different influences on the reliability and the hazard rate in different stage of the service life of the components. Besides, it should be noted that in the wear-out period, the reliability and the hazard rate are quite sensitive to the dispersion of the transverse standard load coefficient. 

In practice, strength is always correlative with the load. It is the function of the occurrence frequency and the magnitude of the load. The assumption that load is independent of strength may cause errors in the calculation of reliability. The models proposed in this paper take the correlation between the load and the strength into consideration. Moreover, they are convenient to use and helpful for the lifecycle management of the components and the systems.
